# Differential Risk of Dementia Between Patients With Atrial Flutter and Atrial Fibrillation: A National Cohort Study

**DOI:** 10.3389/fcvm.2021.787866

**Published:** 2021-11-18

**Authors:** Hui-Ting Wang, Yung-Lung Chen, Yu-Sheng Lin, Huang-Chung Chen, Shaur-Zheng Chong, Shukai Hsueh, Chang-Ming Chung, Mien-Cheng Chen

**Affiliations:** ^1^Department of Emergency, Kaohsiung Chang Gung Memorial Hospital, Kaohsiung, Taiwan; ^2^Division of Cardiology, Department of Internal Medicine, Kaohsiung Chang Gung Memorial Hospital, Kaohsiung, Taiwan; ^3^College of Medicine, Graduate Institute of Clinical Medical Sciences, Chang Gung University, Taoyuan, Taiwan; ^4^Division of Cardiology, Department of Internal Medicine, Chang Gung Memorial Hospital, Chiayi, Taiwan

**Keywords:** atrial fibrillation, atrial flutter, dementia, stroke, CHA_2_DS_2_-VASc score

## Abstract

**Objectives:** Atrial fibrillation (AF) is linked to an increased risk of stroke and dementia. Atrial flutter (AFL) is also linked to an increased risk of stroke but at a different level of risk as compared to AF. Little is known about the difference in the risk of dementia between AF and AFL. This study aims to investigate whether the risk of dementia is different between AF and AFL.

**Methods:** Patients with newly diagnosed AF and AFL during 2001–2013 were retrieved from Taiwan's National Health Insurance Research Database. Patients with incomplete demographic data, aged <20 years, history of valvular surgery, rheumatic heart disease, hyperthyroidism, and history of dementia were excluded. The incidence of new-onset dementia was set as the primary outcome and analyzed in patients with AF and AFL after propensity score matching (PSM).

**Results:** A total of 232,425 and 7,569 patients with AF and AFL, respectively, were eligible for analysis. After 4:1 PSM, we included 30,276 and 7,569 patients with AF and AFL, respectively, for analysis. Additionally, patients with AF (*n* = 29,187) and AFL (*n* = 451) who received oral anticoagulants were enrolled for comparison. The risk of dementia was higher in patients with AF compared with patients with AFL (subdistribution hazard ratio (SHR) = 1.52, 95% CI 1.39–1.66; *p* < 0.0001) before PSM and remained higher in patients with AF (SHR = 1.14, 95% CI 1.04–1.25; *p* = 0.0064) after PSM. The risk of dementia was higher in patients with AF without previous history of stroke after PSM but the risk did not differ between patients with AF and AFL with previous history of stroke. Among patients who received oral anticoagulants, the cumulative incidences of dementia were significantly higher in patients with AF than in patients with AFL before and after PSM (all *P* < 0.05).

**Conclusions:** This study found that, among patients without history of stroke, the risk of dementia was higher in patients with AF than in patients with AFL, and CHA_2_DS_2_-VASc score might be useful for risk stratification of dementia between patients with AF and AFL.

## Introduction

Atrial fibrillation (AF) had been reported to be linked to an increased risk of ischemic stroke, thromboembolism, heart failure, myocardial infarction, and death (1, 2). Moreover, there is increasing evidence that AF is also a risk factor for cognitive decline and dementia, which might be independent of ischemic stroke (3–6). Atrial flutter (AFL) and AF share the common risk factors and therefore, should contribute to similar clinical events. Accordingly, clinical guidelines recommend AFL should be treated as AF in terms of anticoagulation to prevent stroke and systemic thromboembolism (7). However, our previous study showed that the incidence of ischemic stroke was significantly higher in the AF cohort than in the AFL cohort at a CHA_2_DS_2_-VASc score [Heart failure, Hypertension, Age ≥75 (doubled), Diabetes, Stroke (doubled), Vascular disease, Age 65–74, and Sex category (female)] ≥1 (8). Therefore, differential risk of ischemic stroke exists between AF and AFL. There is no study to examine the risk of dementia between patients with AFL and AF. We hypothesized that patients with AF had a higher risk of dementia than patients with AFL independent of the previous history of stroke. Accordingly, we conducted this large population-based national cohort study to evaluate the incidence of dementia in patients with AF and AFL.

## Materials and Methods

### Data Source

Taiwan's National Health Insurance started in 1995 and covers 99.5% (23 million) of the residents in Taiwan. The National Health Insurance Research Database (NHIRD) provides the data of all inpatient and outpatient services, diagnoses, emergency room visits, prescriptions, examinations, operations, and expenditures and are updated biannually. The diseases were diagnosed using the International Classification of Diseases, ninth revision, Clinical Modification (ICD-9-CM) and version 2001 codes. This study was approved by the Institutional Review Board of Chang Gung Memorial Hospital (202100865B1). The dataset used in this study was held by the Taiwan Ministry of Health and Welfare (MOHW). Any researcher interested in accessing this dataset can submit an application form to the MOHW requesting access. (Email: stcarol-wu@mohw.gov.tw).

### Study Patients

**Figure 1:**
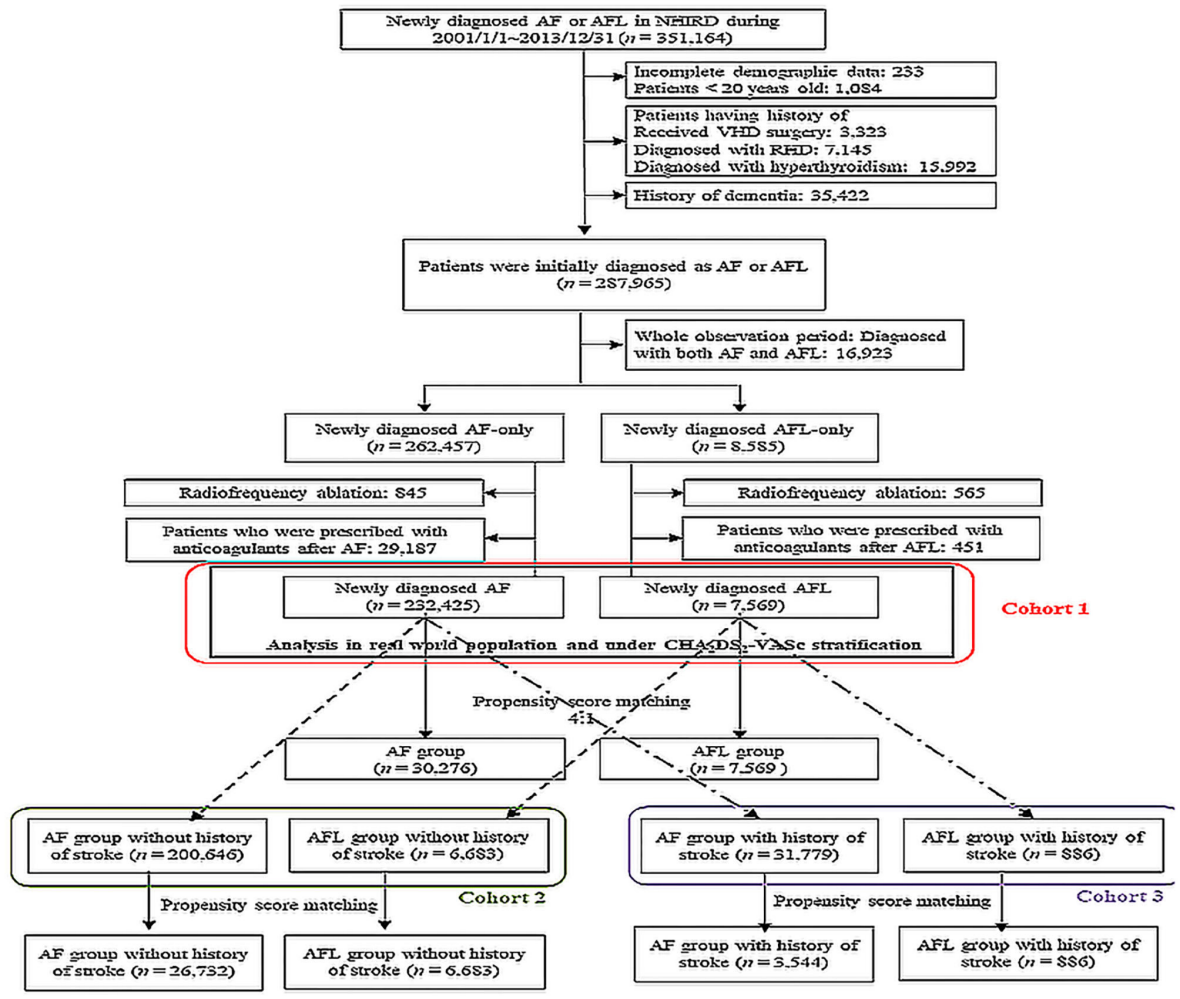
Flow chart of the patient selection. AF, atrial fibrillation; AFL, atrial flutter; NHIRD, National Health Insurance Research Database; RHD, rheumatic heart disease; VHD, valvular heart disease.

Electronic medical records from the NHIRD between January 1, 2001 and December 31, 2013 were retrieved for patients with a discharge diagnosis or at least two consecutive outpatient clinic diagnoses of AF (ICD-9-CM: 427.31) and AFL (ICD-9-CM: 427.32). The time when AF or AFL was first diagnosed was assigned as the index date. The coverage period of our database was from 1997 to 2013; therefore, we excluded patients who were diagnosed with both AF and AFL between 1997 and 2000. Furthermore, we excluded patients who had incomplete demographic data (<0.1%), aged <20 years, history of valvular surgery, rheumatic heart disease, hyperthyroidism, and history of dementia (Figure 1). The remaining patients were categorized into two groups as newly diagnosed AF or newly diagnosed AFL without history of dementia. Furthermore, we excluded those patients who received radiofrequency ablation because these factors might also affect the risk of stroke and dementia.

### Study Design

The primary outcome of dementia was analyzed after propensity score matching (PSM) between AF and AFL population in three different cohorts. In cohort 1, patients with AF (*n* = 232,425) and AFL (*n* = 7,569) were eligible population after serial excluding criteria. Cohort 2 [patients with AF (*n* = 200,646) and AFL (*n* = 6,683)] enrolled those patients without any history of ischemic stroke in cohort 1, while cohort 3 [patients with AF (*n* = 31,779) and AFL (*n* = 886)] enrolled those patients with a history of ischemic stroke in cohort 1 (Figure 1). The PSM was performed with 4:1 ratio under adjusting covariates including age, sex, comorbidities, and medications between AF and AFL cohorts. Furthermore, the primary outcome was also analyzed by stratifying the groups by CHA_2_DS_2_-VASc score in cohort 1 (Figure 1).

### Study Outcome, Covariates, and Follow-Up

The primary outcome was dementia, which was defined when the diagnosis was made during hospitalization or ≥2 consecutive clinic visits. The diagnostic code of dementia was validated in previous NHIRD studies (9, 10). The first date when dementia was coded during follow-up was assigned as the date of the endpoint. Each patient in the three cohorts was followed up until the date of dementia occurrence, death, or December 31, 2013, whichever occurred first.

The covariates included comorbidities, namely hypertension, diabetes mellitus, dyslipidemia, ischemic heart disease, peripheral arterial disease, chronic obstructive pulmonary disease, renal function status, abnormal liver function, gout, systemic thromboembolism, myocardial infarction, stroke, and heart failure, and medications.

These comorbidities were ascertained according to the ICD-9-CM codes combined with medication use. The patients with systemic thromboembolism, myocardial infarction, stroke, and heart failure were defined as having any inpatient diagnosis before the index date. Most of these diagnostic codes were validated in previous NHIRD studies (11–15). Similarly, data on medication usage were retrieved on claim-based data of the previous year.

### Ascertainment of AF/AFL and CHA_2_DS_2_-VASc Score

All patients who had AF or AFL diagnosis defined according to the diagnosis made at least once during hospitalization or ≥2 consecutive clinic visits. The accuracy of AF or AFL diagnosis using ICD-9-CM code in the NHIRD has been confirmed in previous studies (16, 17).

Because CHA_2_DS_2_-VASc score has been reported to be useful for risk stratification regarding ischemic stroke and dementia among patients with AF and AFL (18–21), we stratified patients using CHA_2_DS_2_-VASc score to compare the risk of dementia between patients with AF and AFL before and after PSM. Comorbidities in CHA_2_DS_2_-VASc score were ascertained according to the ICD-9-CM codes combined with medication use and the diagnostic codes were validated in previous NHIRD studies (12–15).

### Statistical Analysis

The propensity score matching was performed with 4:1 ratio in patients with AF and AFL. The covariates in the propensity score calculation were the age, gender, 12 comorbidities, 10 types of medication, and index date. The matching was processed using a greedy nearest neighbor algorithm with a caliper of 0.2 times the SD of the logit of the propensity score. The quality of matching was assessed using the standardized mean difference (SMD) between the two groups after matching, where a value <0.1 indicated a negligible difference (Table 1). The risk of dementia was compared between patients with AF and AFL before and after PSM. The risk of dementia between patients with AF and AFL was also compared after stratification by CHA_2_DS_2_-VASc score and previous history of stroke. A *P* < 0.05 was considered statistically significant. No adjustment for multiple testing (multiplicity) was performed in this study. All statistical analyses were performed using commercial software [Statistical Analysis System (SAS) V.9.4], including the “*psmatch*” procedure for PSM, “*phreg*” procedure for survival analysis, and “%*cif* ” macro for generating a cumulative incidence function through Fine and Gray's method.

**Table 1:** Baseline characteristics of the atrial fibrillation and atrial flutter groups before and after propensity score matching.

**Variables**	**All patients**			**Propensity score matched**	
	**AFL** ** (*n* = 7,569)**	**AF** ** (*n* = 232,425)**	***P*-value**	**AF** ** (*n* = 30,276)**	***P*-value**
Age (years; mean ± SD)	67.41 ± 6.0	72.21 ± 3.3	<0.0001	67.31 ± 5.7	0.6889
Age group			<0.0001		0.725
<65 years	2,841 (37.5%)	59,556 (25.6%)		11,449 (37.8%)	
65~74 years	1,883 (24.9%)	59,783 (25.7%)		7,399 (24.4%)	
≥ 75 years	2,845 (37.6%)	113,086 (48.7%)		11,428 (37.7%)	
Gender			<0.0001		0.6485
Male	4,689 (62.0%)	131,832 (56.7%)		18,842 (62.2%)	
Female	2,880 (38.0%)	100,593 (43.3%)		11,434 (37.8%)	
Comorbidities					
Hypertension	4,199 (55.5%)	139,437 (60.0%)	<0.0001	16,672 (55.1%)	0.5216
Diabetes Mellitus	1,463 (19.3%)	44,672 (19.2%)	0.813	5,834 (19.3%)	0.9066
Ischemic heart disease	2,546 (33.6%)	86,362 (37.2%)	<0.0001	10,111 (33.4%)	0.6909
Heart failure	901 (11.9%)	29,841 (12.8%)	0.0166	3,539 (11.7%)	0.6037
Dyslipidemia	1,086 (14.3%)	30,650 (13.2%)	0.0033	4,189 (13.8%)	0.25
Gout	753 (9.9%)	24,095 (10.4%)	0.2398	3,037 (100%)	0.8305
Chronic obstructive pulmonary disease	1,360 (18.0%)	45,954 (19.8%)	0.0001	5,419 (17.9%)	0.8881
Peripheral arterial disease	321 (4.2%)	11,007 (4.7%)	0.0458	1,242 (4.1%)	0.5875
Renal status			0.0137		0.717
Non-chronic kidney disease	6,384 (84.3%)	198,371 (85.3%)		25,649 (84.7%)	
Chronic kidney disease without dialysis	911 (12.0%)	26,833 (11.5%)		3,549 (11.7%)	
Chronic kidney disease with dialysis	274 (3.6%)	7,221 (3.1%)		1,078 (3.6%)	
Immune disease	147 (1.9%)	3,935 (1.7%)	0.0991	562 (1.9%)	0.6221
Abnormal liver function	904 (11.9%)	26,151 (11.3%)	0.061	3,603 (11.9%)	0.9178
Malignancy	683 (9.0%)	17,722 (7.6%)	<0.0001	2,811 (9.3%)	0.483
History of disease					
Prior stroke or systemic thromboembolism	977 (12.9%)	34,401 (14.8%)	<0.0001	3,928 (13.0%)	0.8784
Prior stroke	886 (11.7%)	31,779 (13.7%)	<0.0001	3,556 (11.7%)	0.9237
Old myocardial infarction	425 (5.6%)	10,307 (4.4%)	<0.0001	1,661 (5.5%)	0.6605
Medications					
ACEi/ARB	2,445 (32.3%)	91,378 (39.3%)	<0.0001	9,795 (32.4%)	0.9343
Calcium channel blockers	1,667 (22.0%)	58,046 (25.0%)	<0.0001	6,672 (22.0%)	0.9802
ß-blockers	2,549 (33.7%)	75,683 (32.6%)	0.0418	9,531 (31.5%)	0.0002
Dipeptidyl peptidase 4 inhibitors	153 (2.0%)	4,518 (1.9%)	0.6308	568 (1.9%)	0.4081
Statins	832 (11.0%)	25,522 (11.0%)	0.975	3,095 (10.2%)	0.0496
Biguanides	697 (9.2%)	21,906 (9.4%)	0.5259	2,756 (9.1%)	0.7752
Sulfonylurea	758 (10.0%)	24,341 (10.5%)	0.2	3,004 (9.9%)	0.8099
Thiazolidinedione	106 (1.4%)	3,160 (1.4%)	0.7626	388 (1.3%)	0.415
Insulin	260 (3.4%)	7,366 (3.2%)	0.1944	946 (3.1%)	0.169
Antiplatelet	2,479 (32.8%)	104,861 (45.1%)	<0.0001	9,968 (32.9%)	0.776

*ACEi, angiotensin converting enzyme inhibitors; AF, atrial fibrillation; AFL, atrial flutter; ARB, angiotensin II receptor blockers*.

### Patient and Public Involvement

Since this study is a retrospective cohort study based on a national insurance database, in no part or stage of the research were the patients/public involved.

## Results

### Study Population

We identified a total of 351,164 patients newly diagnosed with AF or AFL during 2001–2013 in the NHIRD. After exclusions, 232,425 patients with AF (aged 72.2 ±13.3 years) and 7,569 patients with AFL (aged 67.41 ± 6.0 years) without receiving oral anticoagulants were eligible for analysis in the study cohort 1 (Table 1). The distribution of comorbidities was similar between the patients with AF and AFL, except there was a higher prevalence of hypertension, ischemic heart disease, and history of thromboembolism/ischemic stroke in patients with AF compared with patients with AFL. After 4:1 ratio of PSM using all variables in Table 1 and there was a good balance between the patients with AF and AFL, except for ß-blockers (right panel of Table 1), 30,276 patients with AF (aged 67.3 ±15.7 years) and 7,569 patients with AFL were analyzed for comparison of the risk of dementia.

Moreover, we further categorized the original 232,425 patients with AF and 7,569 patients with AFL in the cohort 1 into cohorts 2 and 3 according to history without (cohort 2) or with stroke (cohort 3) and performed the additional PSM in each cohort. After PSM, there were 26,732 patients with AF and 6,683 patients with AFL in the cohort 2, and 3,544 patients with AF and 886 patients with AFL in the cohort 3 (Figure 1).

### Difference in the Risk of Dementia and Ischemic Stroke Between Patients With AF and AFL Before and After PSM (Cohort 1)

**Figure 2:**
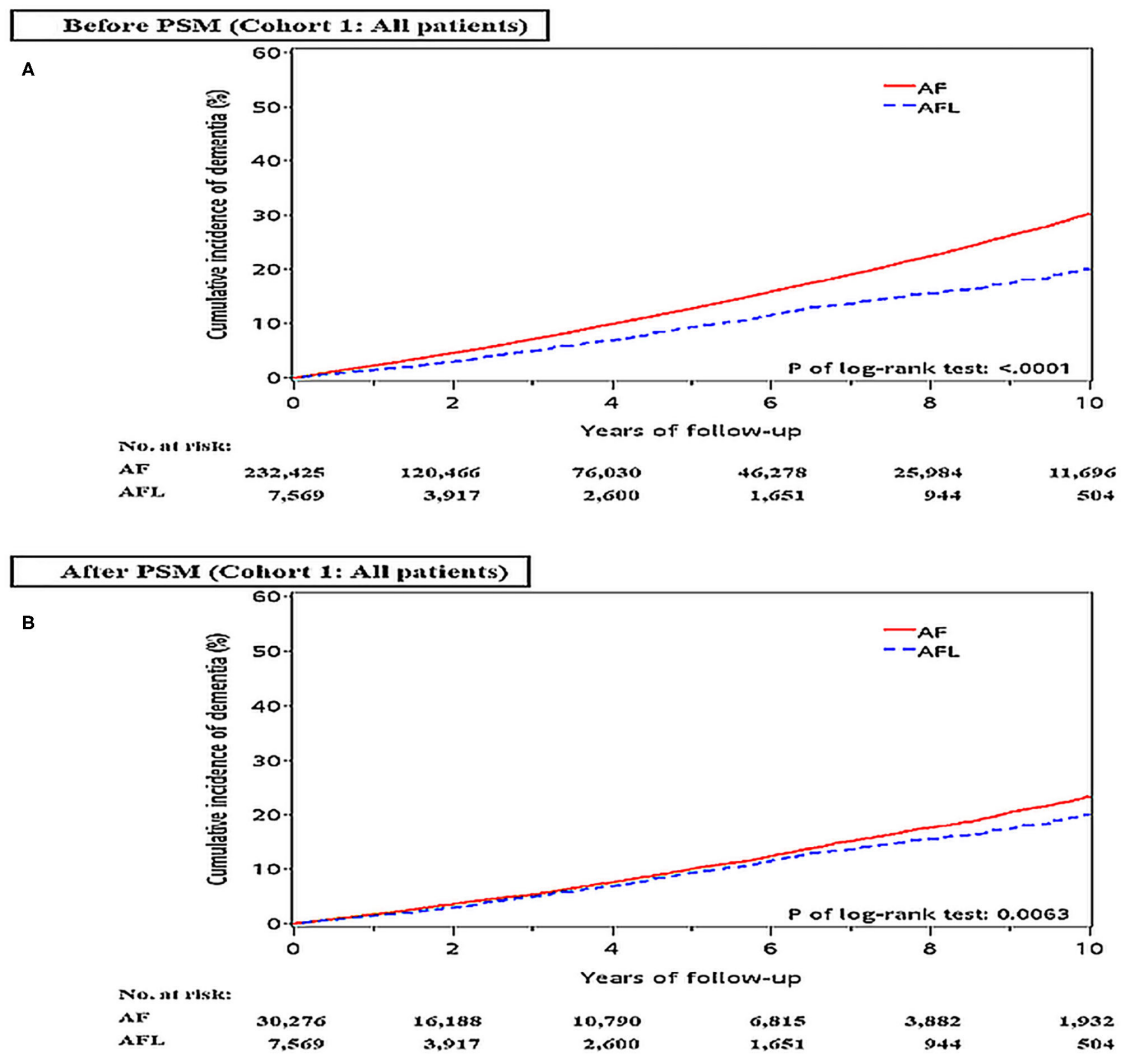
Cumulative incidences of dementia at the end of follow-up between the AF and AFL groups in the whole cohort (cohort 1) before **(A)** and after **(B)** PSM. AF, atrial fibrillation; AFL, atrial flutter; PSM, propensity score matching.

Before PSM, the incidence densities of dementia (hazard ratio [HR], 1.52; 95% CI, 1.39–1.66; *P* < 0.0001) and ischemic stroke (HR, 2.15; 95% CI, 1.92–2.42; *P* < 0.0001) were higher in patients with AF than in patients with AFL (Table 2). After PSM, the incidence densities of dementia (HR, 1.14; 95% CI, 1.04–1.25; *P* = 0.0064) and ischemic stroke (HR, 1.76; 95% CI, 1.56–1.98; *P* < 0.0001) were still higher in patients with AF than in patients with AFL (Table 2). The cumulative incidences of dementia in patients with AF and AFL before and after PSM were shown in Figures 2A,B.

**Table 2:** Clinical outcomes between the patients with atrial fibrillation and atrial flutter not receiving warfarin therapy before and after PSM.

**Variable**	**Before PSM**			**After PSM**		
	**AF (*n* = 232,425)**	**AFL (*n* = 7,569)**	***P*-value**	**AF (*n* = 30,276)**	**AFL (*n* = 7,569)**	***P*-value**
Dementia						
Number of events, *n* (%)	22,833 (9.82)	521 (6.88)		2,435 (8.04)	521 (6.88)	
Incidence density§	3.04 (3.00–3.08)	2.05 (1.87–2.22)		2.33 (2.24–2.43)	2.05 (1.87–2.22)	
Hazard ratio (95% CI)	1.52 (1.39–1.66)	Reference	<0.0001	1.14 (1.04–1.25)	Reference	0.0064
Ischemic stroke						
Number of events, *n* (%)	18,475 (7.95)	297 (3.92)		2,095 (6.92)	297 (3.92)	
Incidence density§	2.54 (2.50–2.58)	1.18 (1.04–1.31)		2.07 (1.98–2.16)	1.18 (1.04–1.31)	
Hazard ratio (95% CI)	2.15 (1.92–2.42)	Reference	<0.0001	1.76 (1.56–1.98)	Reference	<0.0001

*AF, atrial fibrillation; AFL, atrial flutter; PSM, propensity score matching*.

*§ Incidence density: number of events per 100 person-years*.

### Difference in the Risk of Dementia and Ischemic Stroke Between Patients With AF and AFL Without Previous History of Stroke (Cohort 2)

There were 200,646 patients with AF without previous history of stroke and 6,683 patients with AFL without previous history of stroke (Table 3, Figure 1). After 4:1 PSM, 26,732 patients with AF and 6,683 patients with AFL were well balanced, except for ß-blockers (Table 3). Before PSM, the incidence densities of dementia (HR, 1.54; 95% CI, 1.40–1.69; *P* < 0.0001) and ischemic stroke (HR, 2.32; 95% CI, 2.04–2.64; *P* < 0.0001) were higher in patients with AF than in patients with AFL (Table 4). After PSM, the incidence densities of dementia (HR, 1.15; 95% CI, 1.03–1.27; *P* = 0.0098) and ischemic stroke (HR, 1.82; 95% CI, 1.59–2.09; *P* < 0.0001) were higher in patients with AF than in patients with AFL (Table 4).

**Table 3:** Baseline characteristics of the atrial fibrillation and atrial flutter groups before and after PSM (without history of stroke).

**Variables**	**Without history of stroke patients**			**Propensity score matched**	
	**AFL (*n* = 6,683)**	**AF (*n* = 200,646)**	***P*-value**	**AF (*n* = 26,732)**	***P*-value**
Age (years; mean ± SD)	66.41 ± 6.3	71.51 ± 3.6	<0.0001	66.51 ± 6.0	0.8808
Age group			<0.0001		0.7103
<65 years	2,694 (40.3%)	55,494 (27.7%)		10,751 (40.2%)	
65~74 years	1,640 (24.5%)	51,904 (25.9%)		6,457 (24.2%)	
≥ 75 years	2,349 (35.1%)	93,248 (46.5%)		9,524 (35.6%)	
Gender			<0.0001		0.6932
Male	4,130 (61.8%)	114,261 (56.9%)		16,590 (62.1%)	
Female	2,553 (38.2%)	86,385 (43.1%)		10,142 (37.9%)	
Comorbidities					
Hypertension	3,576 (53.5%)	116,639 (58.1%)	<0.0001	14,458 (54.1%)	0.398
Diabetes Mellitus	1,179 (17.6%)	35,701 (17.8%)	0.7504	4,790 (17.9%)	0.5972
Ischemic heart disease	2,168 (32.4%)	72,738 (36.3%)	<0.0001	8,717 (32.6%)	0.7928
Heart failure	705 (10.5%)	22,725 (11.3%)	0.0485	2,883 (10.8%)	0.5778
Dyslipidemia	918 (13.7%)	25,282 (12.6%)	0.006	3,670 (13.7%)	0.9873
Gout	662 (9.9%)	20,528 (10.2%)	0.3879	2,645 (9.9%)	0.9781
Chronic obstructive pulmonary disease	1,134 (17.0%)	37,762 (18.8%)	0.0001	4,489 (16.8%)	0.7311
Peripheral arterial disease	245 (3.7%)	8,668 (4.3%)	0.0095	960 (3.6%)	0.7692
Renal status			0.0191		0.809
Non-chronic kidney disease	5,702 (85.3%)	173,283 (86.4%)		22,725 (85.0%)	
Chronic kidney disease without dialysis	758 (11.3%)	21,634 (10.8%)		3,088 (11.6%)	
Chronic kidney disease with dialysis	223 (3.3%)	5,729 (2.9%)		919 (3.4%)	
Immune disease	132 (2.0%)	3,421 (1.7%)	0.0941	520 (1.9%)	0.8743
Abnormal liver function	812 (12.2%)	22,840 (11.4%)	0.0523	3,299 (12.3%)	0.6711
Malignancy	606 (9.1%)	15,458 (7.7%)	<0.0001	2,422 (9.1%)	0.9848
History of disease					
Prior systemic thromboembolism	91 (1.4%)	2,622 (1.3%)	0.6977	390 (1.5%)	0.5505
Old myocardial infarction	323 (4.8%)	7,614 (3.8%)	<0.0001	1,348 (5.0%)	0.4822
Medications					
ACEi/ARB	2,104 (31.5%)	78,575 (39.2%)	<0.0001	8,499 (31.8%)	0.6257
Calcium channel blockers	1,419 (21.2%)	48,585 (24.2%)	<0.0001	5,858 (21.9%)	0.2278
ß-blockers	2,300 (34.4%)	67,200 (33.5%)	0.1155	8,644 (32.3%)	0.0012
Dipeptidyl peptidase 4 inhibitors	129 (1.9%)	3,748 (1.9%)	0.7115	458 (1.7%)	0.2272
Statins	736 (11.0%)	21,750 (10.8%)	0.6545	2,732 (10.2%)	0.0573
Biguanides	594 (8.9%)	18,483 (9.2%)	0.368	2,332 (8.7%)	0.6703
Sulfonylurea	645 (9.7%)	20,391 (10.2%)	0.1732	2,567 (9.6%)	0.904
Thiazolidinedione	95 (1.4%)	2,665 (1.3%)	0.5126	348 (1.3%)	0.4441
Insulin	188 (2.8%)	5,525 (2.8%)	0.77	798 (3.0%)	0.4572
Antiplatelet	2,118 (31.7%)	89,775 (44.7%)	<0.0001	8,524 (31.9%)	0.7601

*ACEi, angiotensin converting enzyme inhibitors; AF, atrial fibrillation; AFL, atrial flutter; ARB, angiotensin II receptor blockers*.

**Table 4:** Clinical outcomes between the patients with atrial fibrillation and atrial flutter before and after PSM (without history of stroke).

**Variable**	**Before PSM**			**After PSM**		
	**AF (*n* = 200,646)**	**AFL (*n* = 6,683)**	***P*-value**	**AF (*n* = 26,732)**	**AFL (*n* = 6,683)**	***P*-value**
Dementia						
Number of events, *n* (%)	18,693 (9.32)	431 (6.45)		1,983 (7.42)	431 (6.45)	
Incidence density§	2.77 (2.73–2.81)	1.85 (1.67–2.02)		2.11 (2.02–2.20)	1.85 (1.67–2.02)	
Hazard ratio (95% CI)	1.54 (1.40–1.69)	Reference	<0.0001	1.15 (1.03–1.27)	Reference	0.0098
Ischemic stroke						
Number of events, *n* (%)	15,119 (7.54)	232 (3.47)		1,661 (6.21)	232 (3.47)	
Incidence density§	2.31 (2.28–2.35)	1.00 (0.87–1.13)		1.82 (1.73–1.91)	1.00 (0.87–1.13)	
Hazard ratio (95% CI)	2.32 (2.04–2.64)	Reference	<0.0001	1.82 (1.59–2.09)	Reference	<0.0001

*AF, atrial fibrillation; AFL, atrial flutter; PSM, propensity score matching*.

*§ Incidence density: number of events per 100 person-years*.

**Figure 3:**
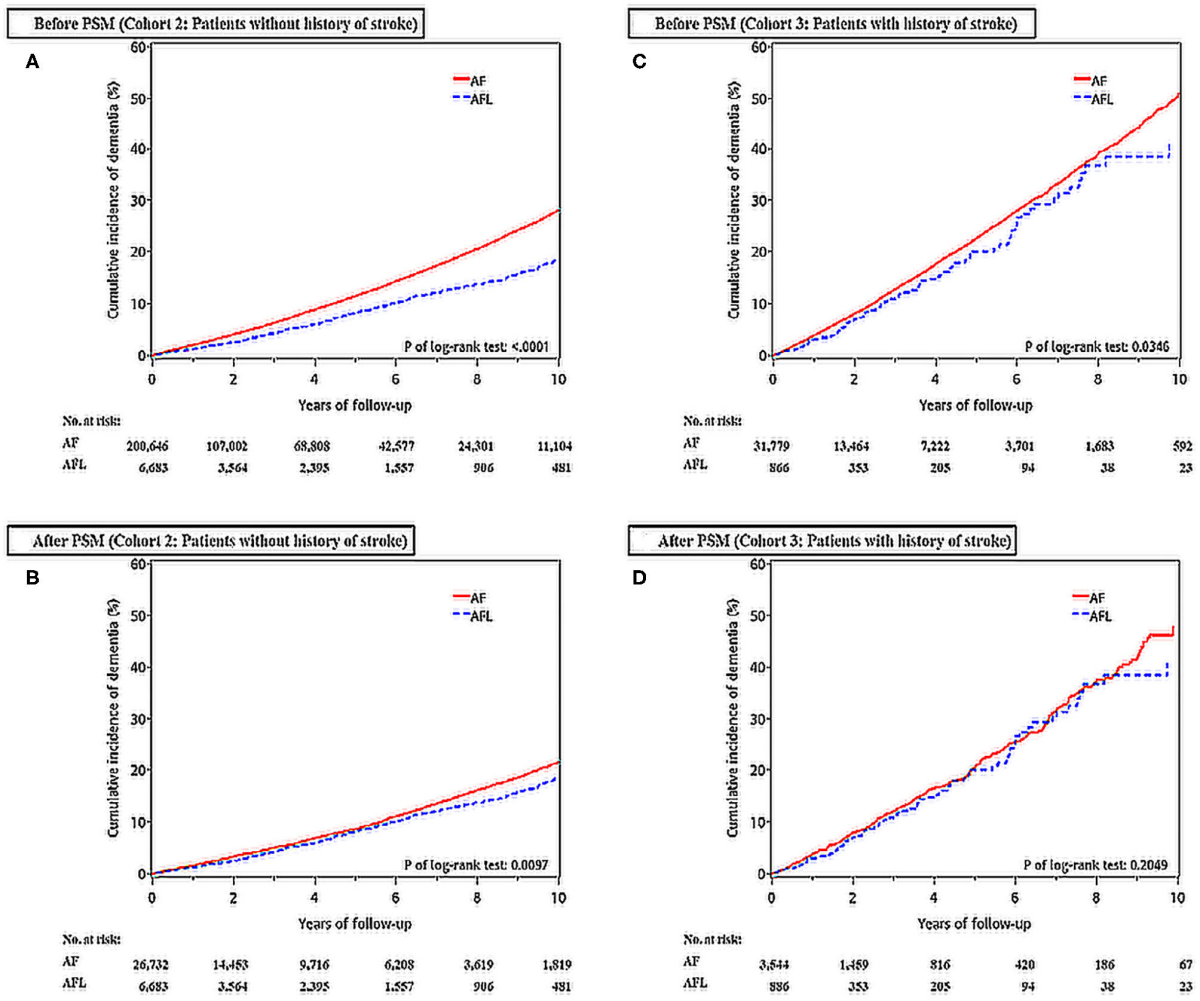
Cumulative incidences of dementia at the end of follow-up between the AF and AFL groups before and after PSM in those patients without (cohort 2) and with (cohort 3) history of stroke. **(A)** Patients without history of stroke before PSM; **(B)** Patients without history of stroke after PSM; **(C)** Patients with history of stroke before PSM; **(D)** Patients with history of stroke after PSM.

The cumulative incidences of dementia in patients with AF and AFL without previous history of stroke before and after PSM were shown in Figures 3A,B.

### Difference in the Risk of Dementia and Recurrent Ischemic Stroke Between Patients With AF and AFL With Previous History of Stroke (Cohort 3)

There were 31,779 patients with AF with a history of stroke and 886 patients with AFL with a history of stroke (Table 5, Figure 1). After 4:1 PSM, 3,544 patients with AF and 886 patients with AFL were well balanced (Table 5). Before PSM, the incidence densities of dementia (HR, 1.25; 95% CI, 1.02–1.54; *P* = 0.0351) and recurrent ischemic stroke (HR, 1.39; 95% CI, 1.09–1.78; *P* = 0.0083) were higher in patients with AF than in patients with AFL (Table 6). After PSM, the incidence density of dementia did not differ between patients with AF and AFL (HR, 1.16; 95% CI, 0.92–1.45; *P* = 0.2058) (Table 6). However, the incidence density of recurrent ischemic stroke (HR, 1.40; 95% CI, 1.08–1.83; *P* = 0.0118) was higher in patients with AF than in patients with AFL (Table 6).

**Table 5:** Baseline characteristics of the atrial fibrillation and atrial flutter groups before and after PSM (with history of stroke).

**Variables**	**With history of stroke patients**			**Propensity score matched**	
	**AFL (*n* = 886)**	**AF (*n* = 31,779)**	***P*-value**	**AF (*n* = 3,544)**	***P*-value**
Age (years; mean ± SD)	75.01 ± 1.1	76.61 ± 0.0	<0.0001	75.01 ± 0.7	0.9391
Age group			0.0001		0.9949
<65 years	147 (16.6%)	4,062 (12.8%)		589 (16.6%)	
65~74 years	243 (27.4%)	7,879 (24.8%)		966 (27.3%)	
≥ 75 years	496 (56.0%)	19,838 (62.4%)		1,989 (56.1%)	
Gender			<0.0001		0.9504
Male	559 (63.1%)	17,571 (55.3%)		2,232 (63.0%)	
Female	327 (36.9%)	14,208 (44.7%)		1,312 (37.0%)	
Comorbidities					
Hypertension	623 (70.3%)	22,798 (71.7%)	0.3536	2,478 (69.9%)	0.8185
Diabetes Mellitus	284 (32.1%)	8,971 (28.2%)	0.0127	1,167 (32.9%)	0.6197
Ischemic heart disease	378 (42.7%)	13,624 (42.9%)	0.9021	1,523 (43.0%)	0.8674
Heart failure	196 (22.1%)	7,116 (22.4%)	0.849	769 (21.7%)	0.7849
Dyslipidemia	168 (19.0%)	5,368 (16.9%)	0.1053	697 (19.7%)	0.6357
Gout	91 (10.3%)	3,567 (11.2%)	0.3747	360 (10.2%)	0.9208
Chronic obstructive pulmonary disease	226 (25.5%)	8,192 (25.8%)	0.8561	899 (25.4%)	0.9312
Peripheral arterial disease	76 (8.6%)	2,339 (7.4%)	0.1719	302 (8.5%)	0.9571
Renal status			0.233		0.7953
Non-chronic kidney disease	682 (77.0%)	25,088 (78.9%)		2,691 (75.9%)	
Chronic kidney disease without dialysis	153 (17.3%)	5,199 (16.4%)		645 (18.2%)	
Chronic kidney disease with dialysis	51 (5.8%)	1,492 (4.7%)		208 (5.9%)	
Immune disease	15 (1.7%)	514 (1.6%)	0.8604	65 (1.8%)	0.7779
Abnormal liver function	92 (10.4%)	3,311 (10.4%)	0.9731	345 (9.7%)	0.5623
Malignancy	77 (8.7%)	2,264 (7.1%)	0.0746	301 (8.5%)	0.8507
History of disease					
Old myocardial infarction	102 (11.5%)	2,693 (8.5%)	0.0014	423 (11.9%)	0.7274
Medications					
ACEi/ARB	341 (38.5%)	12,803 (40.3%)	0.2812	1,319 (37.2%)	0.4849
Calcium channel blockers	248 (28%)	9,461 (29.8%)	0.2528	1,039 (29.3%)	0.4367
ß-blockers	249 (28.1%)	8,483 (26.7%)	0.3496	932 (26.3%)	0.2769
Dipeptidyl peptidase 4 inhibitors	24 (2.7%)	770 (2.4%)	0.5858	106 (3.0%)	0.6562
Statins	96 (10.8%)	3,772 (11.9%)	0.3473	460 (13.0%)	0.0848
Biguanides	103 (11.6%)	3,423 (10.8%)	0.4191	422 (11.9%)	0.8162
Sulfonylurea	113 (12.8%)	3,950 (12.4%)	0.7729	532 (15.0%)	0.0884
Thiazolidinedione	11 (1.2%)	495 (1.6%)	0.4524	71 (2.0%)	0.1324
Insulin	72 (8.1%)	1,841 (5.8%)	0.0035	234 (6.6%)	0.1097
Antiplatelet	361 (40.7%)	15,086 (47.5%)	<0.0001	1,461 (41.2%)	0.7952

*ACEi, angiotensin converting enzyme inhibitors; AF, atrial fibrillation; AFL, atrial flutter; ARB, angiotensin II receptor blockers*.

**Table 6:** Clinical outcomes between the patients with atrial fibrillation and atrial flutter before and after PSM (with history of stroke).

**Variable**	**Before PSM**			**After PSM**		
	**AF (*n* = 31,779)**	**AFL (*n* = 886)**	***P*-value**	**AF (*n* = 3,544)**	**AFL (*n* = 886)**	***P*-value**
Dementia						
Number of events, n (%)	4,140 (13.03)	90 (10.16)		427 (12.05)	90 (10.16)	
Incidence density§	5.33 (5.17–5.49)	4.31 (3.42–5.20)		4.97 (4.50–5.44)	4.31 (3.42–5.20)	
Hazard ratio (95% CI)	1.25 (1.02–1.54)	Reference	0.0351	1.16 (0.92–1.45)	Reference	0.2058
Ischemic stroke						
Number of events, n (%)	3,356 (10.56)	65 (7.34)		368 (10.38)	65 (7.34)	
Incidence density§	4.59 (4.43–4.74)	3.27 (2.48–4.07)		4.60 (4.13–5.07)	3.27 (2.48–4.07)	
Hazard ratio (95% CI)	1.39 (1.09–1.78)	Reference	0.0083	1.40 (1.08–1.83)	Reference	0.0118

*AF, atrial fibrillation; AFL, atrial flutter; PSM, propensity score matching*.

*§ Incidence density: number of events per 100 person-years*.

The cumulative incidences of dementia in patients with AF and AFL with a history of stroke before and after PSM were shown in Figures 3C,D.

### The Risk of Dementia Stratified According to CHA_2_DS_2_-VASc Score Between Patients With AF and AFL (Cohort 1)

**Figure 4:**
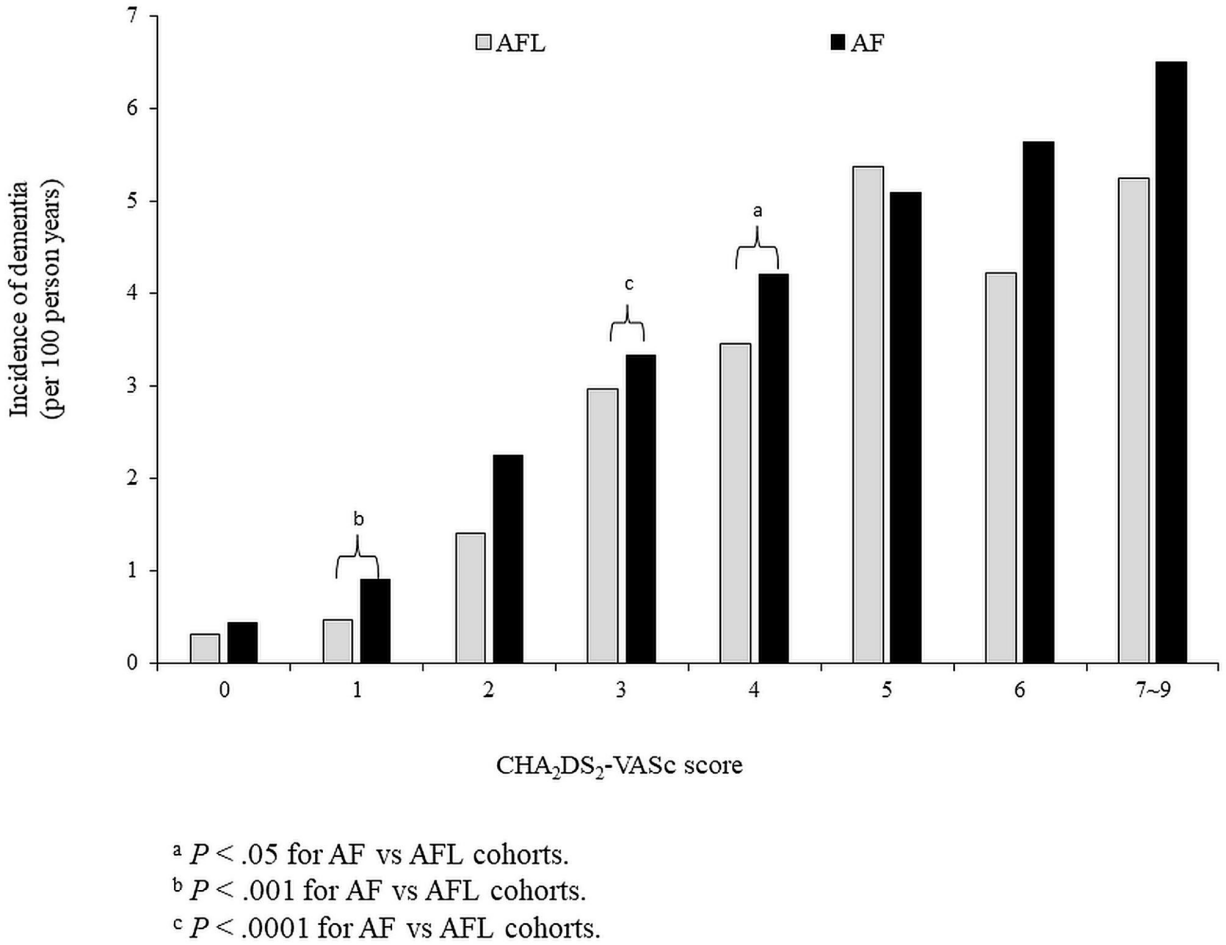
The risk of dementia in the whole cohort (cohort 1) was stratified according to CHA_2_DS_2_-VASc score before propensity score matching. AF, atrial fibrillation; AFL, atrial flutter.

**Figure 5:**
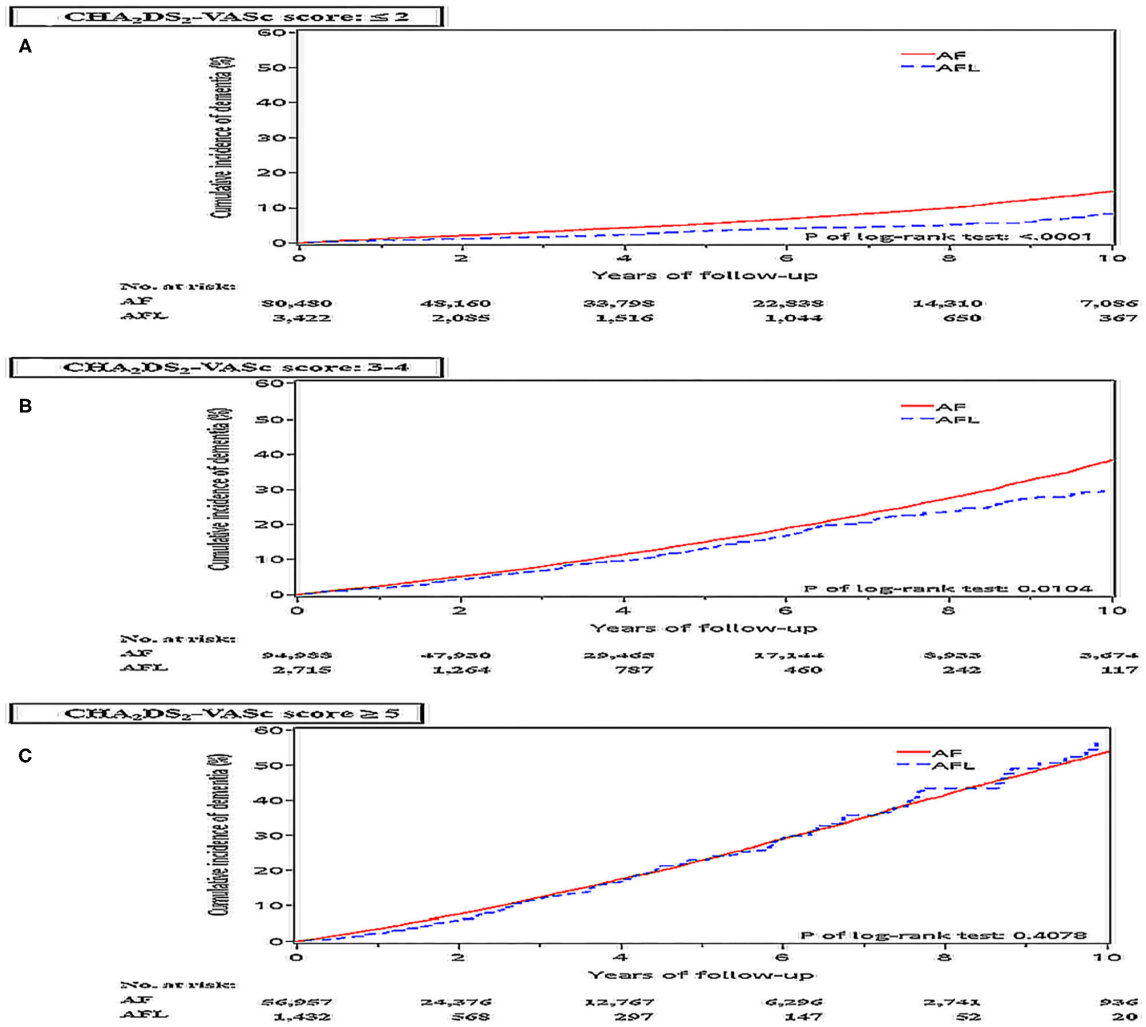
Cumulative incidences of dementia at the end of follow up between the AF and AFL groups stratified by CHA_2_DS_2_-VASc score in the whole cohort (cohort 1) after PSM. **(A)** CHA_2_DS_2_-VASc score ≤ 2; **(B)** CHA_2_DS_2_-VASc score: 3-4; **(C)** CHA_2_DS_2_-VASc score ≥ 5.

The risk of dementia in the cohort 1 was stratified according to CHA_2_DS_2_-VASc score. Before PSM, the incidence density of dementia was higher in patients with AF than in patients with AFL across CHA_2_DS_2_-VASc scores of 1–4 and 6–9 (Figure 4). After PSM, the cumulative incidences of dementia in patients with AF and AFL stratified according to different CHA_2_DS_2_-VASc scores were shown in Figure 5, and the cumulative incidences of dementia were significantly higher in patients with AF than patients with AFL in patients with CHA_2_DS_2_-VASc score ≤ 2 (*P* < 0.0001) and patients with CHA_2_DS_2_-VASc score between 3 and 4 (*P* = 0.0104), but not in patients with CHA_2_DS_2_-VASc score ≥ 5.

### Difference in the Risk of Dementia and Ischemic Stroke Between Patients With AF and AFL Receiving Oral Anticoagulants Therapy Before and After PSM

Because the usage of oral anticoagulant might affect the risk of dementia and ischemic stroke in patients with AF and AFL, we identified and analyzed the risk of dementia and ischemic stroke between patients newly diagnosed with AF (*n* = 29,187) and AFL (*n* = 451) who received oral anticoagulants (mainly vitamin K oral anticoagulant) in another dataset during the same study period in the NHIRD.

**Figure 6:**
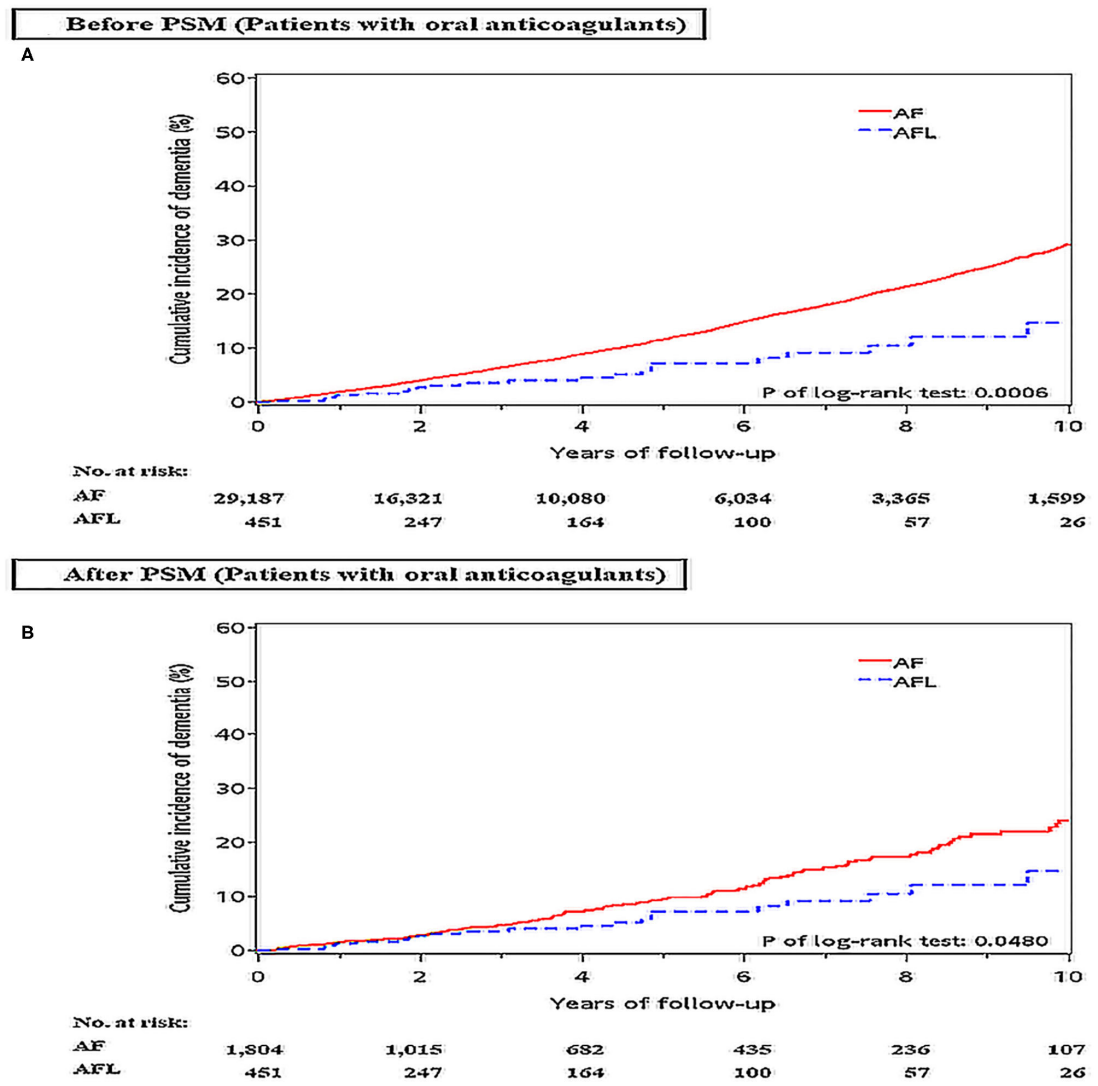
Cumulative incidences of dementia at the end of follow-up between patients with AF and AFL receiving oral anticoagulants therapy before **(A)** and after **(B)** PSM. AF: atrial fibrillation; AFL, atrial flutter; PSM, propensity score matching.

Before PSM, the incidence densities of dementia (HR, 2.05; 95% CI, 1.35–3.11; *P* = 0.0008) and ischemic stroke (HR, 2.95; 95% CI, 1.86–4.62; *P* < 0.0001) were higher in patients with AF than in patients with AFL (Table 7). After PSM, the incidence density of dementia (HR, 1.57; 95% CI, 1.00–2.45; *P* = 0.0501) was higher with borderline significance in patients with AF than in patients with AFL, and the incidence density of ischemic stroke (HR, 2.54; 95% CI, 1.56–4.12; *P* = 0.0002) was significantly higher in patients with AF than in patients with AFL (Supplemental Table 5). The cumulative incidences of dementia in patients with AF and AFL before and after PSM were shown in Figures 6A,B and the cumulative incidences of dementia were significantly higher in patients with AF than in patients with AFL before and after PSM.

**Table 7:** Clinical outcomes between the patients with atrial fibrillation and atrial flutter who received oral anticoagulants before and after PSM.

**Variable**	**Before PSM**			**After PSM**		
	**AF (*n* = 29,187)**	**AFL (*n* = 451)**	***P*-value**	**AF (*n* = 1,840)**	**AFL (*n* = 451)**	***P*-value**
Dementia						
Number of events, n (%)	2,830 (9.70)	22 (4.88)		144 (7.98)	22 (4.88)	
Incidence density§	2.81 (2.71–2.91)	1.39 (0.81–1.97)		2.20 (1.84–2.56)	1.39 (0.81–1.97)	
Hazard ratio (95% CI)	2.05 (1.35–3.11)	Reference	0.0008	1.57 (1.00–2.45)	Reference	0.0501
Ischemic stroke						
Number of events, n (%)	3,255 (11.15)	18 (3.99)		181 (10.03)	18 (3.99)	
Incidence density§	3.43 (3.31–3.54)	1.16 (0.62–1.70)		2.95 (2.52–3.38)	1.16 (0.62–1.70)	
Hazard ratio (95% CI)	2.95 (1.86–4.62)	Reference	<0.0001	2.54 (1.56–4.12)	Reference	0.0002

*AF, atrial fibrillation; AFL, atrial flutter; PSM, propensity score matching*.

*§ Incidence density: number of events per 100 person-years*.

## Discussions

The main findings of this study were (1) among patients without oral anticoagulants, the incidence densities of dementia and ischemic stroke were significantly higher in patients with AF than in patients with AFL before and after PSM; (2) among patients without oral anticoagulants, the incidence densities of dementia and ischemic stroke were significantly higher in patients with AF than in patients with AFL among patients without previous history of stroke before and after PSM; (3) among patients without oral anticoagulants, the incidence density of dementia did not differ between patients with AF and AFL among patients with previous history of stroke after PSM; (4) among patients without oral anticoagulants, the cumulative incidence of dementia was significantly higher in patients with AF than patients with AFL only in patients with CHA_2_DS_2_-VASc score ≤ 4; (5) among patients who received oral anticoagulants, the cumulative incidences of dementia were significantly higher in patients with AF than in patients with AFL before and after PSM.

Atrial fibrillation provides five-fold increase in the risk of stroke and AF is associated with more severe ischemic strokes than emboli from carotid disease (18, 22). It has been reported that AF is associated with cognitive dysfunction and dementia, and may increase the risk of developing dementia (4, 23–26). Several pathophysiologic mechanisms have been proposed to explain the complex association between AF and dementia, which include cerebral hypoperfusion, vascular inflammation, cerebral small vessel disease, microbleeds, brain atrophy, and AF-related ischemic stroke (3, 5, 23, 27, 28). AF and AFL share some common risk factors for cognitive dysfunction or dementia. When more cardiovascular disease risks coexist, the risk of dementia in patients with AF will increase significantly. CHA_2_DS_2_-VASc score has been reported to be useful for risk stratification regarding ischemic stroke and dementia among patients with AF and AFL (18–21, 29). Study conducted by Chou et al. reported that CHADS_2_ score had a good prediction of vascular dementia in patients with AF and AFL (3). The higher the score, the higher the number of patients suffering from dementia. CHA_2_DS_2_-VASc score is composed of chronic diseases and also a history of stroke. Therefore, not only AF or AFL alone might contribute to the development of dementia, but all the comorbidities do. In this study, we also showed that the risk of dementia is higher in both AF and AFL cohort with higher CHA_2_DS_2_-VASc scores. Based on our and other studies, the CHA_2_DS_2_-VASc score might be a useful risk scoring system for predicting future risk of development of vascular dementia and for risk stratification in patients with AF and AFL at risk of developing dementia (18). In this study, patients with AF were older than patients with AFL and had more comorbidities, including prior stroke, hypertension, ischemic heart disease, heart failure, and a higher average CHA_2_DS_2_-VASc score. All of these differences may contribute to a higher incidence of dementia in patients with AF than in patients with AFL before adjusting the comorbidities in this study. However, the incidence of dementia is still higher in patients with AF than in patients with AFL even after adjusting the comorbidities and medications by PSM, especially in those patients without previous history of stroke. Therefore, differential risk of dementia existed between AF and AFL patients without previous history of stroke.

Although AF and AFL share some common risk factors and can both be risk stratified by CHA_2_DS_2_-VASc score, the pathophysiological mechanisms of AF and AFL are different. The initiation and maintenance of AF usually involved the pulmonary veins and depended on multiple chaotic rotors in the arrhythmogenic substrate in the left or right atrium for maintenance. In contrast, the maintenance of AFL depended on a single reentry circuit, such as single reentry circuit in the right atrium of a typical AFL. Our previous study showed that the incidences of ischemic stroke, hospitalization for heart failure, and all-cause mortality are different between AF and AFL, which might be related to the different pathophysiological mechanisms between AF and AFL (8, 30). Although the risk of dementia is higher in patients with AF than in patients with AFL in this present study, further studies should be conducted to explore the pathophysiologic mechanisms of differential risk of dementia between patients with AF and AFL.

The previous studies showed that there were conflicting data regarding the therapeutic effect of oral anticoagulants on the risk of dementia in patients with AF (31). Although oral anticoagulants might decrease the risk of dementia by reducing the risk of silent infarct and microembolism, microhemorrhages, especially due to supratherapeutic range of anticoagulation, may have implications for the mechanism of dementia (32, 33). In contrast, non-vitamin K oral anticoagulants may significantly reduce the occurrence of dementia (33–35). This may explain the discrepancy of oral anticoagulants on the risk of dementia in patients with AF. Our studies showed that among patients who received oral anticoagulants (mainly vitamin K oral anticoagulant), the cumulative incidence of dementia was significantly higher in patients with AF than in patients with AFL. Accordingly, the usage of oral anticoagulants may not affect the differential risk of dementia between patients with AF and AFL. Further prospective randomized studies are warranted to validate our findings and to investigate the effect of different oral anticoagulants on the risk of dementia in patients with AF and AFL.

Several limitations are associated with epidemiologic data from the NHIRD. First, using ICD-9-CM codes for patient selection may result in some missing cases due to incorrect coding. Some patients who had dementia with indistinct symptoms may be also missed due to the unavailability of clinical characteristics or image studies. Second, certain misclassification of disease leading to the miscalculation of the CHA_2_DS_2_-VASc score may have occurred due to the retrospective nature of the study. However, the highly diagnostic accuracy of comorbidities in CHA_2_DS_2_-VASc score has been validated in previous studies. Third, we could not categorize the mechanisms of AFL into typical or atypical AFL and right or left AFL by ICD-9-CM codes. The clinical outcome may be different between patients with typical and atypical AFL and also between patients with left and right AFL. Further investigation should be conducted to clarify this issue. Fourth, AF was developed in around 30% AFL during follow-up according to previous studies (36). Although we have excluded those patients who have concomitant AF and AFL, there is still the possibility that some patients with AFL who developed AF during follow-up might not be diagnosed in the clinical follow-up. Despite this concern, we showed that the incidence density of dementia was significantly higher in patients with AF than in patients with AFL without previous history of stroke. Fifth, this study showed that among patients who received oral anticoagulants (mainly vitamin K oral anticoagulants), the cumulative incidence of dementia was significantly higher in patients with AF than in patients with AFL. However, this study did not analyze the effect of non-vitamin K oral anticoagulants on the risk of dementia in patients with AF and AFL. A previous study showed that non-vitamin K oral anticoagulants may lower the risk of dementia in patients with AF (33–35). Further prospective randomized studies are warranted to investigate the effect of different non-vitamin K oral anticoagulants on the risk of dementia in patients with AF and AFL. Sixth, although HAS-BLED score (hypertension, abnormal renal/liver function, stroke, bleeding history or predisposition, labile international normalized ratio, elderly [age ≥65 years], drugs/alcohol concomitantly) and CHA_2_DS_2_-VASc score share many common risk factors, in a real-world population with AF, the CHA_2_DS_2_-VASc and HAS-BLED risk classifications are correlated but not exchangeable (37). Future research should be conducted to investigate the risk of dementia stratified according to HAS-BLED score alone or combined CHA_2_DS_2_-VASc score and HAS-BLED score between patients with AF and AFL. Seventh, although this study used PSM with all known variables to achieve a good balance between patients with AF and AFL, unavailable confounding risk factors might be still present. Finally, this study did not subclassify all subtypes of dementia. There might be a difference in the risk of developing different subtypes of dementia between AF and AFL. Further studies could be conducted to clarify this issue.

## Conclusions

This large nationwide cohort study showed that among patients without oral anticoagulants, the risk of dementia was higher in patients with AF than in patients with AFL. Among patients without oral anticoagulants, the risk of dementia was higher in patients with AF than in patients with AFL with CHA_2_DS_2_-VASc score ≤ 4. Among patients without oral anticoagulants, the risk of dementia was higher in patients with AF than in patients with AFL among patients without previous history of stroke before and after PSM but the risk did not differ between patients with AF and AFL among patients with a previous history of stroke after PSM. Among patients who received oral anticoagulants, the cumulative incidences of dementia were significantly higher in patients with AF than in patients with AFL before and after PSM. Our study implicated that CHA_2_DS_2_-VASc score might be useful for risk stratification of dementia between patients with AF and AFL.

## Data Availability Statement

The raw data supporting the conclusions of this article will be made available by the authors, without undue reservation.

## Ethics Statement

The studies involving human participants were reviewed and approved by Institutional Review Board of Chang Gung Memorial Hospital. Written informed consent for participation was not required for this study in accordance with the national legislation and the institutional requirements.

## Author Contributions

C-MC and M-CC led in the conception and design of the study, revised the draft of the manuscript, and supervised and validated the clinical work and results. H-TW, Y-LC, and Y-SL collected research data, prepared the draft of the manuscript, performed the statistical analysis, and drafted the manuscript. H-CC, S-ZC, and SH organized the collected data. All authors have read and agreed to the published version of the manuscript.

## Conflict of Interest

The authors declare that the research was conducted in the absence of any commercial or financial relationships that could be construed as a potential conflict of interest.

## Publisher's Note

All claims expressed in this article are solely those of the authors and do not necessarily represent those of their affiliated organizations, or those of the publisher, the editors and the reviewers. Any product that may be evaluated in this article, or claim that may be made by its manufacturer, is not guaranteed or endorsed by the publisher.

## References

[1] Freedman B Potpara TS Lip GY. Stroke prevention in atrial fibrillation. Lancet. (2016) 388:806–17. 10.1016/S0140-6736(16)31257-0 27560276

[2] Staerk L Sherer JA Ko D Benjamin EJ Helm RH. Atrial Fibrillation: Epidemiology, pathophysiology, and clinical outcomes. Circ Res. (2017) 120:1501–17. 10.1161/CIRCRESAHA.117.309732 28450367 PMC5500874

[3] Chou RH Chiu CC Huang CC Chan WL Huang PH Chen YC. Prediction of vascular dementia and Alzheimer's disease in patients with atrial fibrillation or atrial flutter using CHADS2 score. J Chin Med Assoc. (2016) 79:470–6. 10.1016/j.jcma.2016.02.007 27234974

[4] Aldrugh S Sardana M Henninger N Saczynski JS McManus DD. Atrial fibrillation, cognition and dementia: A review. J Cardiovasc Electrophysiol. (2017) 28:958–65. 10.1111/jce.13261 28569383 PMC5783292

[5] Chopard R Piazza G Gale SA Campia U Albertsen IE Kim J. Dementia and atrial fibrillation: pathophysiological mechanisms and therapeutic implications. Am J Med. (2018) 131:1408–17. 10.1016/j.amjmed.2018.06.035 30076825

[6] Saglietto A Matta M Gaita F Jacobs V Bunch TJ Anselmino M. Stroke-independent contribution of atrial fibrillation to dementia: a meta-analysis. Open Heart. (2019) 6:e000984. 10.1136/openhrt-2018-000984 31217998 PMC6546265

[7] January CT Wann LS Calkins H Chen LY Cigarroa JE Cleveland JC Jr.. AHA/ACC/HRS focused update of the 2014 AHA/ACC/HRS guideline for the management of patients with atrial fibrillation: a report of the American college of cardiology/American heart association task force on clinical practice guidelines and the heart rhythm society. J Am Coll Cardiol. (2019) 74:104–32. 10.1016/j.jacc.2019.01.011 30703431

[8] Lin YS Chen TH Chi CC Lin MS Tung TH Liu CH. Different implications of heart failure, ischemic stroke, and mortality between nonvalvular atrial fibrillation and atrial flutter-a view from a national cohort study. J Am Heart Assoc. (2017) 6:e006406. 10.1161/JAHA.117.006406 28733435 PMC5586326

[9] Chen CW Lin CC Chen KB Kuo YC Li CY Chung CJ. Increased risk of dementia in people with previous exposure to general anesthesia: a nationwide population-based case-control study. Alzheimers Dement. (2014) 10:196–204. 10.1016/j.jalz.2013.05.1766 23896612

[10] Zhang B Wang HE Bai YM Tsai SJ Su TP Chen TJ. Inflammatory bowel disease is associated with higher dementia risk: a nationwide longitudinal study. Gut. (2021) 70:85–91. 10.1136/gutjnl-2020-320789 32576641

[11] Wu CS Lai MS Gau SS Wang SC Tsai HJ. Concordance between patient self-reports and claims data on clinical diagnoses, medication use, and health system utilization in Taiwan. PLoS ONE. (2014) 9:e112257. 10.1371/journal.pone.0112257 25464005 PMC4251897

[12] Lin CC Lai MS Syu CY Chang SC Tseng FY. Accuracy of diabetes diagnosis in health insurance claims data in Taiwan. J Formos Med Assoc. (2005) 104:157–63. 15818428

[13] Hsieh CY Chen CH Li CY Lai ML. Validating the diagnosis of acute ischemic stroke in a National Health Insurance claims database. J Formos Med Assoc. (2015) 114:254–9. 10.1016/j.jfma.2013.09.009 24140108

[14] Cheng CL Lee CH Chen PS Li YH Lin SJ Yang YH. Validation of acute myocardial infarction cases in the national health insurance research database in taiwan. J Epidemiol. (2014) 24:500–7. 10.2188/jea.JE20140076 25174915 PMC4213225

[15] Chang SL Huang YL Lee MC Hu S Hsiao YC Chang SW. Association of varicose veins with incident venous thromboembolism and peripheral artery disease. JAMA. (2018) 319:807–17. 10.1001/jama.2018.0246 29486040 PMC5838574

[16] Lin LJ Cheng MH Lee CH Wung DC Cheng CL Kao Yang YH. Compliance with antithrombotic prescribing guidelines for patients with atrial fibrillation–a nationwide descriptive study in Taiwan. Clin Ther. (2008) 30:1726–36. 10.1016/j.clinthera.2008.09.010 18840379

[17] Chang CH Lee YC Tsai CT Chang SN Chung YH Lin MS. Continuation of statin therapy and a decreased risk of atrial fibrillation/flutter in patients with and without chronic kidney disease. Atherosclerosis. (2014) 232:224–30. 10.1016/j.atherosclerosis.2013.11.036 24401243

[18] Joundi RA Cipriano LE Sposato LA Saposnik G Stroke outcomes research working G. Ischemic stroke risk in patients with atrial fibrillation and CHA_2_DS_2_-VASc score of 1: systematic review and meta-analysis. Stroke. (2016) 47:1364–7. 10.1161/STROKEAHA.115.012609 27026630

[19] Al-Kawaz M Omran SS Parikh NS Elkind MSV Soliman EZ Kamel H. Comparative risks of ischemic stroke in atrial flutter versus atrial fibrillation. J Stroke Cerebrovasc Dis. (2018) 27:839–44. 10.1016/j.jstrokecerebrovasdis.2017.10.025 29223550

[20] Chao TF Fauchier L. Stroke prevention in patients with atrial flutter: many questions still unanswered. Europace. (2019) 21:186–7. 10.1093/europace/euy197 30202888

[21] Graves KG May HT Jacobs V Knowlton KU Muhlestein JB Lappe DL. CHA_2_DS_2_-VASc scores and Intermountain Mortality Risk Scores for the joint risk stratification of dementia among patients with atrial fibrillation. Heart Rhythm. (2019) 16:3–9. 10.1016/j.hrthm.2018.10.018 30611392

[22] Pistoia F Sacco S Tiseo C Degan D Ornello R Carolei A. The epidemiology of atrial fibrillation and stroke. Cardiol Clin. (2016) 34:255–68. 10.1016/j.ccl.2015.12.002 27150174

[23] Dietzel J Haeusler KG Endres M. Does atrial fibrillation cause cognitive decline and dementia? Europace. (2018) 20:408–19. 10.1093/europace/eux031 28387847

[24] Field TS Weijs B Curcio A Giustozzi M Sudikas S Katholing A. Incident atrial fibrillation, dementia and the role of anticoagulation: a population-based cohort study. Thromb Haemost. (2019) 119:981–91. 10.1055/s-0039-1683429 30919384

[25] Kim D Yang PS Yu HT Kim TH Jang E Sung JH. Risk of dementia in stroke-free patients diagnosed with atrial fibrillation: data from a population-based cohort. Eur Heart J. (2019) 40:2313–23. 10.1093/eurheartj/ehz386 31212315

[26] Krawczyk M Fridman S Cheng Y Fang J Saposnik G Sposato LA. Atrial fibrillation diagnosed after stroke and dementia risk: cohort study of first-ever ischaemic stroke patients aged 65 or older. Europace. (2019) 21:1793–801. 10.1093/europace/euz237 31531673

[27] Gallinoro E D'Elia S Prozzo D Lioncino M Natale F Golino P. Cognitive function and atrial fibrillation: from the strength of relationship to the dark side of prevention. Is there a contribution from sinus rhythm restoration and maintenance? Medicina (Kaunas). (2019) 55:587. 10.3390/medicina55090587 31540311 PMC6780629

[28] Wandell P Carlsson AC Sundquist J Sundquist K. The association between relevant comorbidities and dementia in patients with atrial fibrillation. Geroscience. (2018). 10.1007/s11357-018-0029-8 29934733 PMC6060202

[29] Bunch TJ. Atrial Fibrillation and Dementia. Circulation. (2020) 142:618–20. 10.1161/CIRCULATIONAHA.120.045866 32804567

[30] Lin YS Chen YL Chen TH Lin MS Liu CH Yang TY. Comparison of clinical outcomes among patients with atrial fibrillation or atrial flutter stratified by CHA_2_DS_2_-VASc score. JAMA Netw Open. (2018) 1:e180941. 10.1001/jamanetworkopen.2018.0941 30646091 PMC6324304

[31] Moffitt P Lane DA Park H O'Connell J Quinn TJ. Thromboprophylaxis in atrial fibrillation and association with cognitive decline: systematic review. Age Ageing. (2016) 45:767–75. 10.1093/ageing/afw104 27496936

[32] Jacobs V Woller SC Stevens S May HT Bair TL Anderson JL. Time outside of therapeutic range in atrial fibrillation patients is associated with long-term risk of dementia. Heart Rhythm. (2014) 11:2206–13. 10.1016/j.hrthm.2014.08.013 25111326

[33] Cheng W Liu W Li B Li D. Relationship of anticoagulant therapy with cognitive impairment among patients with atrial fibrillation: a meta-analysis and systematic review. J Cardiovasc Pharmacol. (2018) 71:380–7. 10.1097/FJC.0000000000000575 29528873

[34] Madhavan M Graff-Radford J Piccini JP Gersh BJ. Cognitive dysfunction in atrial fibrillation. Nat Rev Cardiol. (2018) 15:744–56. 10.1038/s41569-018-0075-z 30275499

[35] Mongkhon P Naser AY Fanning L Tse G Lau WCY Wong ICK. Oral anticoagulants and risk of dementia: A systematic review and meta-analysis of observational studies and randomized controlled trials. Neurosci Biobehav Rev. (2019) 96:1–9. 10.1016/j.neubiorev.2018.10.025 30391408

[36] Chen YL Wang HT Chen HC Liu WH Chong SZ Hsueh SK. Proposed A2C2S2-VASc score for predicting atrial fibrillation development in patients with atrial flutter. Open Heart. (2021) 8:e001478. 10.1136/openhrt-2020-001478 33514633 PMC7849887

[37] Marcucci M Lip GY Nieuwlaat R Pisters R Crijns HJ Iorio A. Stroke and bleeding risk co-distribution in real-world patients with atrial fibrillation: the Euro Heart Survey. Am J Med. (2014) 127:979–86 e2. 10.1016/j.amjmed.2014.05.003 24838192

