# Corrigendum: Differential Risk of Dementia Between Patients With Atrial Flutter and Atrial Fibrillation: A National Cohort Study

**DOI:** 10.3389/fcvm.2021.819771

**Published:** 2021-12-09

**Authors:** Hui-Ting Wang, Yung-Lung Chen, Yu-Sheng Lin, Huang-Chung Chen, Shaur-Zheng Chong, Shukai Hsueh, Chang-Ming Chung, Mien-Cheng Chen

**Affiliations:** ^1^Department of Emergency, Kaohsiung Chang Gung Memorial Hospital, Kaohsiung, Taiwan; ^2^Division of Cardiology, Department of Internal Medicine, Kaohsiung Chang Gung Memorial Hospital, Kaohsiung, Taiwan; ^3^College of Medicine, Graduate Institute of Clinical Medical Sciences, Chang Gung University, Taoyuan, Taiwan; ^4^Division of Cardiology, Department of Internal Medicine, Chang Gung Memorial Hospital, Chiayi, Taiwan

**Keywords:** atrial fibrillation, atrial flutter, dementia, stroke, CHA_2_DS_2_-VASc score

In the original article there was an error with the Author Contributions Statement. The corrected Author Contributions Statement appears below.

Hui-Ting Wang and Yu-Sheng Lin contributed equally as first authors

Chang-Ming Chung and Mien-Cheng Chen contributed equally as corresponding authors

The authors apologize for this error and state that this does not change the scientific conclusions of the article in any way. The original article has been updated.

## Publisher's Note

All claims expressed in this article are solely those of the authors and do not necessarily represent those of their affiliated organizations, or those of the publisher, the editors and the reviewers. Any product that may be evaluated in this article, or claim that may be made by its manufacturer, is not guaranteed or endorsed by the publisher.

